# Curcumin and Andrographis Exhibit Anti-Tumor Effects in Colorectal Cancer via Activation of Ferroptosis and Dual Suppression of Glutathione Peroxidase-4 and Ferroptosis Suppressor Protein-1

**DOI:** 10.3390/ph16030383

**Published:** 2023-03-02

**Authors:** Katsuki Miyazaki, Caiming Xu, Mitsuo Shimada, Ajay Goel

**Affiliations:** 1 Department of Molecular Diagnostics and Experimental Therapeutics, Beckman Research Institute of City of Hope Comprehensive Cancer Center, Duarte, CA 91016, USA; 2 Department of Surgery, Tokushima University, Tokushima 770-0042, Japan; 3 Department of General Surgery, The First Affiliated Hospital of Dalian Medical University, Dalian 116004, China

**Keywords:** curcumin, andrographis, glutathione peroxidase 4, ferroptosis suppressor protein 1, natural products, colorectal cancer

## Abstract

Colorectal cancer (CRC) is the leading cause of cancer-related deaths worldwide. The limitations of current chemotherapeutic drugs in CRC include their toxicity, side effects, and exorbitant costs. To assess these unmet needs in CRC treatment, several naturally occurring compounds, including curcumin and andrographis, have gained increasing attention due to their multi-targeted functionality and safety vs. conventional drugs. In the current study, we revealed that a combination of curcumin and andrographis exhibited superior anti-tumor effects by inhibiting cell proliferation, invasion, colony formation, and inducing apoptosis. Genome-wide transcriptomic expression profiling analysis revealed that curcumin and andrographis activated the ferroptosis pathway. Moreover, we confirmed the gene and protein expression of glutathione peroxidase 4 (GPX-4) and ferroptosis suppressor protein 1 (FSP-1), the two major negative regulators of ferroptosis, were downregulated by this combined treatment. With this regimen, we also observed that intracellular accumulation of reactive oxygen species and lipid peroxides were induced in CRC cells. These cell line findings were validated in patient-derived organoids. In conclusion, our study revealed that combined treatment with curcumin and andrographis exhibited anti-tumorigenic effects in CRC cells through activation of ferroptosis and by dual suppression of GPX-4 and FSP-1, which have significant potential implications for the adjunctive treatment of CRC patients.

## 1. Introduction

Colorectal cancer (CRC) is the third most common malignancy and the second leading cause of cancer-related deaths worldwide [1]. Although population-based screening programs have contributed to an increased number of cases diagnosed with early-stage CRC [2], more than 20% of patients still have distant metastases at the time of diagnosis, and their 5 year survival rates are less than 15% [3,4]. Even among the patients without metastatic disease, ~40% experience recurrence after initial treatment [4]. Although resection can cure a smaller subset of patients with metastatic disease, systemic chemotherapy remains the primary treatment for metastatic and recurrent CRC [4]. Depending on the tumor’s molecular subtype, several chemotherapeutic options are available for patients with metastatic CRC. To prevent drug resistance, most chemotherapeutic regimens use a combination of multiple anti-tumor drugs. However, the acquisition of drug resistance remains one of the primary causes of tumor progression. Moreover, additional limitations of currently used combinatorial therapeutic modalities include drug toxicity, off-target side effects, and exorbitant costs. To overcome these unmet clinical challenges for patients with CRC, there is an imperative need to identify and evaluate other safe and cost-effective modalities that can be used as potential adjunctive and integrative medicine approaches [5,6].

Several naturally occurring, botanical-driven compounds have recently been scientifically interrogated, offering multi-targeted and safer therapeutic options vis-a-vis conventional cancer chemotherapy [5,7,8]. Among these, curcumin, extracted from Curcuma longa, is one of the most studied compounds due to its potent anti-cancer activity in various human cancers [6,7,8]. Curcumin acts as a chemo-sensitizer against various anti-tumor therapies but simultaneously has protective effects on normal organs [8,9,10,11]. We have previously reported the anti-tumor effects of curcumin on CRC via epigenetic changes, including DNA methylation, gene expression, and miRNA expression, all of which can modulate malignancy and the tumor microenvironment [10,12,13,14]. In addition, recent evidence suggests that curcumin’s anti-tumor activity may partly be mediated via ferroptosis [15,16,17].

Ferroptosis is a unique type of programmed cell death process that depends on the intracellular accumulation of iron, followed by enhanced production of reactive oxygen species (ROS) and lipid peroxides, eventually promoting iron-dependent cell death in cancer cells [5,18,19]. Recently, ferroptosis has been considered an endogenous anticancer mechanism and is actively being studied to develop novel therapeutic targets in cancer [5]. In this context, curcumin and other naturally-occurring botanicals have also been shown to be ferroptosis inducers in cancer cells [5,16,20,21,22]. We previously reported that andrographis, an active compound derived from the Chinese herbal medicine andrographis paniculate, exhibited anti-tumor activity in gastric and CRC via the activation of the ferroptosis pathway [21,22,23,24,25]. Based on these studies, data suggest that curcumin and andrographis exhibit an anti-tumor effect in cancer by activating the ferroptosis pathway. However, the detailed mechanism of ferroptosis induced by these botanicals remains unclear. Therefore, we hypothesized that a combination treatment using curcumin and andrographis might synergistically enhance their anti-tumor potential through increased ferroptosis. We also investigated how these compounds mediate key genes involved in the ferroptosis pathway.

Accordingly, the present study revealed that such a combination treatment exhibited promising anti-cancer synergistic effects in CRC cells and patient-derived 3D tumor organoids. Furthermore, genome-wide transcriptomic profiling analysis demonstrated that the combined treatment with curcumin and andrographis resulted in the induction of ferroptosis in CRC cells, which is in part mediated by the dual suppression of glutathione peroxidase 4 (GPX-4) and ferroptosis suppressor protein 1 (FSP-1), the major negative regulators of ferroptosis [26,27,28].

## 2. Results

### 2.1. Curcumin and Andrographis Combined Treatment Exhibited Anti-Tumor Effects in Colon Cancer Cells via Inhibition of Proliferation, Colony Formation, and Induction of Apoptosis

**Figure 1:**
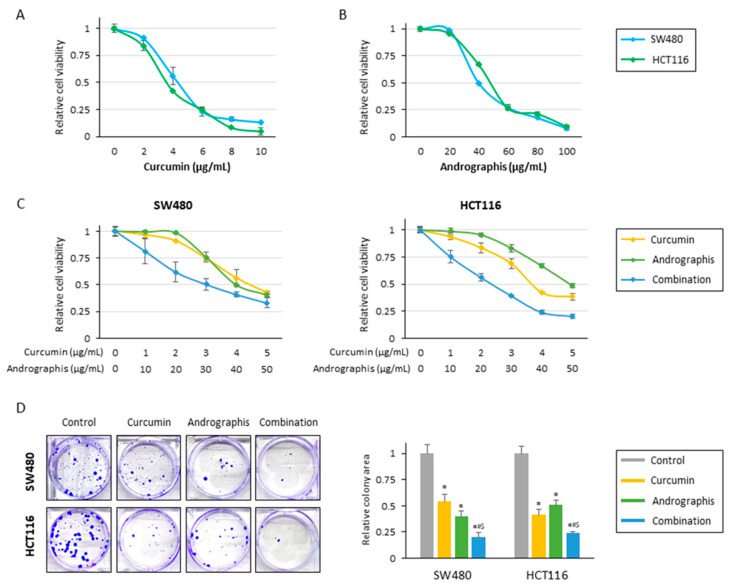
Curcumin and andrographis combinations exhibited an anti-tumor effect in CRC cells. (**A**) Cell viability assay following curcumin treatment for 48 h in SW480 and HCT116. The error bars represent the mean ± SD. (**B**) Cell viability assay following andrographis treatment for 48 h in SW480 and HCT116. The error bars represent the mean ± SD. (**C**) Cell viability assay following curcumin and andrographis combination treatment for 48 h in SW480 and HCT116. Error bars represent the mean ± SD. (**D**) Colony formation assays to assess the clonogenicity of CRC cells following treatment with curcumin, andrographis, and their combinations. The average (column) ± SD is indicated (*; *p*-value < 0.05 vs. control, #; *p*-value < 0.05 vs. curcumin, $; *p*-value < 0.05 vs. andrographis). Cur: curcumin; andro: andrographis.

The current study examined the anti-tumor effects of curcumin, andrographis, and their combination treatment in CRC cells. In line with our previous studies, cell viability assays for individual compounds revealed that curcumin and andrographis exhibited an anti-proliferative effect in SW480 and HCT116 cell lines in a dose-dependent manner (Figure 1A,B). Curcumin’s half-maximal inhibitory concentration (IC_50_) was about 4 μg/mL in both cell lines, and the IC_50_ of andrographis was approximately 40 μg/mL in both cells. From these results, the ratio of curcumin to andrographis in combination treatment was determined to be 1:10 (Figure 1C). In both cell lines, the combined treatment with curcumin and andrographis showed the most potent antiproliferative effects, and the IC_50s_ of the combined treatment were curcumin, 3.0 μg/mL, and andrographis, 30.0 μg/mL, in SW480, and curcumin, 2.4 μg/mL, and andrographis, 24.0 μg/mL, in HCT116. We used these concentrations for all subsequent experiments.

The cell viability experiments showed that curcumin and andrographis exhibited anti-proliferative effects; their combination treatment revealed the most potent anti-proliferative effects. Next, we evaluated the impact of these compounds on the malignant potential of CRC cells. Regarding colony formation ability, individual treatments with curcumin and andrographis significantly reduced colonies in both SW480 (reduction rate; curcumin: 46%, and andrographis: 60%) and HCT116 (reduction rate; curcumin: 59%, and andrographis: 49%; Figure 1D). However, the combination treatment was significantly more potent and reduced more than 75% of colonies in both cell lines, and this effect was more pronounced than the efficacy of the individual compounds (SW480: curcumin vs. combination, *p* = 0.02; andrographis vs. combination, *p* < 0.05; HCT116: curcumin vs. combination, *p* < 0.05; andrographis vs. combination, *p* = 0.02; Figure 1D).

**Figure 2:**
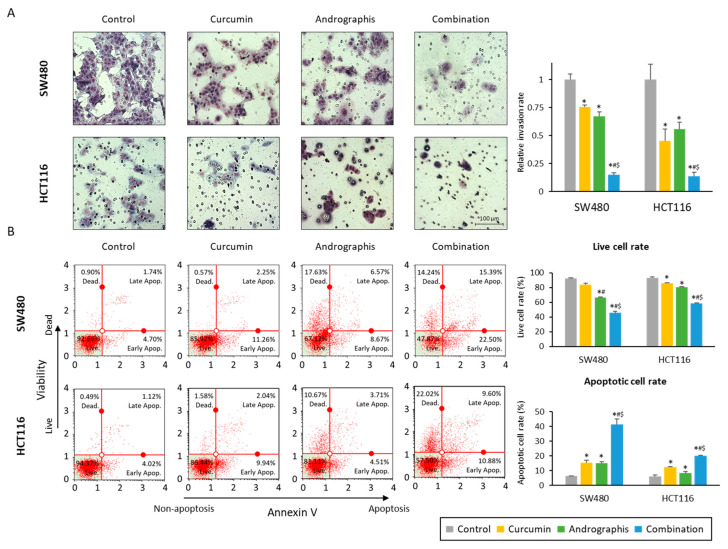
Curcumin and andrographis combination treatment revealed anti-tumor effects via reduction of invasion and induction of apoptosis. (**A**) Invasion assay following treatment with curcumin, andrographis, and their combination for 48 h in SW480 and HCT116. Scale bar = 100 μm. The number of invading cells was counted randomly at three fields on the membrane, and relative invasion ratios were calculated. The average (column) ± SD is indicated (*; *p*-value < 0.05 vs. control, #; *p*-value < 0.05 vs. curcumin, $; *p*-value < 0.05 vs. andrographis). (**B**) Representative images of cells undergoing apoptosis that stained for the annexin V assay in SW480 and HCT116. The average (column) ± SD is indicated (*; *p*-value < 0.05 vs. control, #; *p*-value < 0.05 vs. curcumin, $; *p*-value < 0.05 vs. andrographis). Cur: curcumin; andro: andrographis.

We also evaluated the invasion ability of cancer cells, and the relative invasion of the cells was significantly decreased in both curcumin (76% in SW480, 45% in HCT116) and andrographis (67% in SW480, 56% in HCT116) treated cell lines (Figure 2A). More importantly, the combination treatment further dramatically reduced invaded cells to 15% in SW480 and 14% in HCT116 cells (Figure 2A). The apoptotic cell population after treatment was examined by an annexin V assay, and we observed a reduction in the number of viable cell rates and an increase in the fraction of the apoptotic cell population with each compound and their combination (Figure 2B). As was the case for other assays, the combination treatment led to a significant increase in the number of apoptotic cells vs. the treatment with curcumin or andrographis individually in both SW480 (curcumin vs. combination, 15.2% vs. 41.4%, *p* < 0.01; andrographis vs. combination, 15.0% vs. 41.1%, *p* < 0.01) and HCT116 cell lines (curcumin vs. combination, 12.3% vs. 20.1%, *p* < 0.01; andrographis vs. combination, 8.5% vs. 20.1%, *p* < 0.01; Figure 2B). These results highlight that a combination treatment exhibited more promising anti-tumor effects in CRC compared to the individual treatment with curcumin or andrographis.

### 2.2. Combined Treatment with Curcumin and Andrographis: Activating the Ferroptosis Pathway by Dual Suppression of GPX-4 and FSP-1 in Colon Cancer Cells

**Figure 3:**
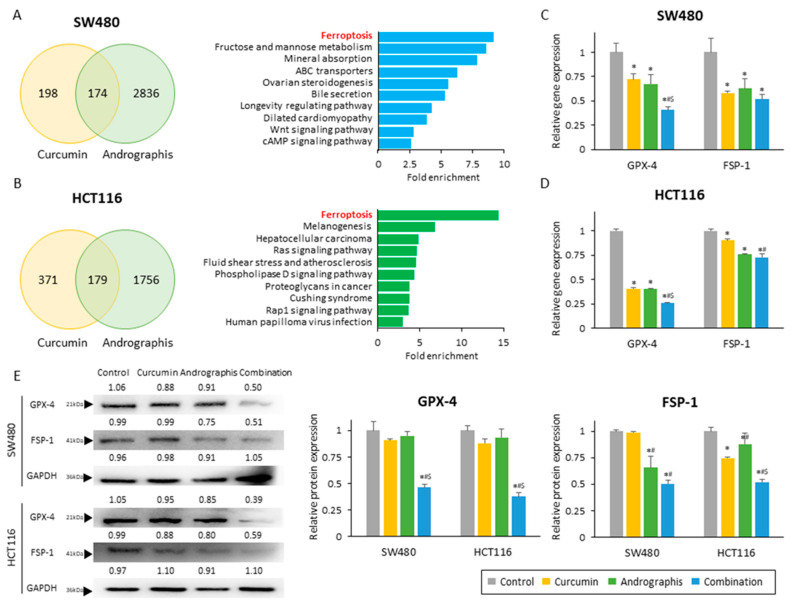
Curcumin and andrographis activated the ferroptosis pathway via dual suppression of GPX-4 and FSP-1. (**A**) Venn diagram of differentially expressed genes (|log FC| > 0.5, *p* < 0.05) and top 10 modulated pathways following treatment with curcumin and andrographis in SW480. (**B**) Venn diagram of differentially expressed genes (|log FC| > 0.5, *p* < 0.05) and top 10 modulated pathways following treatment with curcumin and andrographis in HCT116. (**C**) Relative gene expression of GPX-4 and FSP-1 in SW480 after treatment. (*; *p*-value < 0.05 vs. control, #; *p*-value < 0.05 vs. curcumin, $; *p*-value < 0.05 vs. andrographis). (**D**) Relative gene expression of GPX-4 and FSP-1 in HCT116 after treatment. (*; *p*-value < 0.05 vs. control, #; *p*-value < 0.05 vs. curcumin, $; *p*-value < 0.05 vs. andrographis). (**E**) Western blotting of GPX-4 and FSP-1 protein expression in SW480 and HCT116 following treatment with curcumin, andrographis, and their combination. GAPDH was used as an internal control. The average (column) ± SD is indicated (*; *p*-value < 0.05 vs. control, #; *p*-value < 0.05 vs. curcumin, $; *p*-value < 0.05 vs. andrographis). Cur: curcumin, andro: andrographis, GPX-4: glutathione peroxidase 4, FSP-1: ferroptosis suppressor protein 1.

Next, to identify the molecular pathways responsible for the anti-cancer effects of curcumin and andrographis, we performed genome-wide expression profiling in SW480 and HCT116 cells treated with these two botanicals. First, we identified all differentially expressed genes (|log FC| > 0.5 and *p* < 0.05) between untreated and treated cells and selected the common genes that were differentially expressed among curcumin and andrographis treatments (Figure 3A,B; left chart). We noticed 174 genes in SW480 and 179 genes in HCT 116 cells that were commonly expressed following treatment with these two compounds. Second, we undertook the gene ontology analysis of these differentially expressed genes using KEGG pathway analysis. Interestingly, the pathway with the highest fold enrichment in two cell lines was common, the ferroptosis pathway (fold enrichment: 9.2 in SW480 and 14.4 in HCT116; Figure 3A,B; exemplary chart). These analyses suggested that curcumin and andrographis can modulate and activate the ferroptosis pathway, and this molecular pathway might be one of the primary mediators of their anti-cancer efficacy in the combination treatment group.

To strengthen our findings from genome-wide expression profiling analysis, we next evaluated the gene and protein expression of GPX-4 and FSP-1, which are well known and independent suppressors of ferroptosis. We observed that the gene expression of GPX-4 was significantly reduced by both curcumin and andrographis treatment individually in both SW480 (relative gene expression: 72.0% in curcumin; 67.3% in andrographis) and HCT116 cells (relative gene expression: 40.5% in curcumin; 40.1% in andrographis; Figure 3C,D). More importantly, the combination treatment significantly reduced GPX-4 gene expression by 40.6% in SW480 (control vs. combination, *p* > 0.01; curcumin vs. combination, *p* = 0.01; andrographis vs. combination, *p* = 0.03) and 25.8% in HCT116 (control vs. combination, *p* > 0.01; curcumin vs. combination, *p* < 0.01; and andrographis vs. combination, *p* < 0.01; Figure 3C,D), which were significantly higher reduction rates compared to the individual compounds. Likewise, the gene expression of FSP-1 was also significantly reduced by individual botanicals and the combinatorial treatment with both compounds (Figure 3C,D). Although we did not observe a statistically significant reduction between individual compounds and their combination, the conjunctive treatment still demonstrated the highest reduction rates of 48% in SW480 and 28% in HCT116 (Figure 3C,D). Next, we performed Western blotting to examine the protein expression of these two targets, where the combined treatment revealed the most potent suppression effects on both targets (Figure 3E). Interestingly, individual compounds could reduce the protein expression only up to 12.0% for the GPX-4 expression (reduction rate, SW480: 9.2% by curcumin, 4.9% by andrographis; HCT116: 12.0% by curcumin, 6.6% by andrographis) and 33.5% for the FSP-1 protein (reduction rate, SW480: 1.2% by curcumin, 34.3% by andrographis; HCT116: 25.5% by curcumin, 12.1% by andrographis; Figure 3E). However, the combined treatment with both compounds was more remarkable and resulted in a significantly decreased expression of GPX-4 by 53.7% in SW480 (control vs. combination, *p* < 0.01; curcumin vs. combination, *p* < 0.01; andrographis vs. combination, *p* < 0.01) and by 62.1% in HCT116 cells (control vs. combination, *p* < 0.01; curcumin vs. combination, *p* < 0.01; andrographis vs. combination, *p* < 0.01; Figure 3E). Moreover, this combination reduced the FSP-1 expression by 49.4% in SW480 (control vs. combination, *p* < 0.01; curcumin vs. combination, *p* < 0.01; andrographis vs. combination, *p* < 0.01) and 47.9% in HCT116 (control vs. combination, *p* < 0.01; curcumin vs. combination, *p* < 0.01; andrographis vs. combination, *p* < 0.01; Figure 3E).

Together with these results, we revealed that the synergistic curcumin and andrographis combination treatment could activate the ferroptosis pathway via suppression of two major negative regulators of ferroptosis, GPX-4 and FSP-1.

### 2.3. Curcumin and Andrographis Synergistically Induced Accumulation of Reactive Oxygen Species following Lipid Peroxidation in CRC Cells

**Figure 4:**
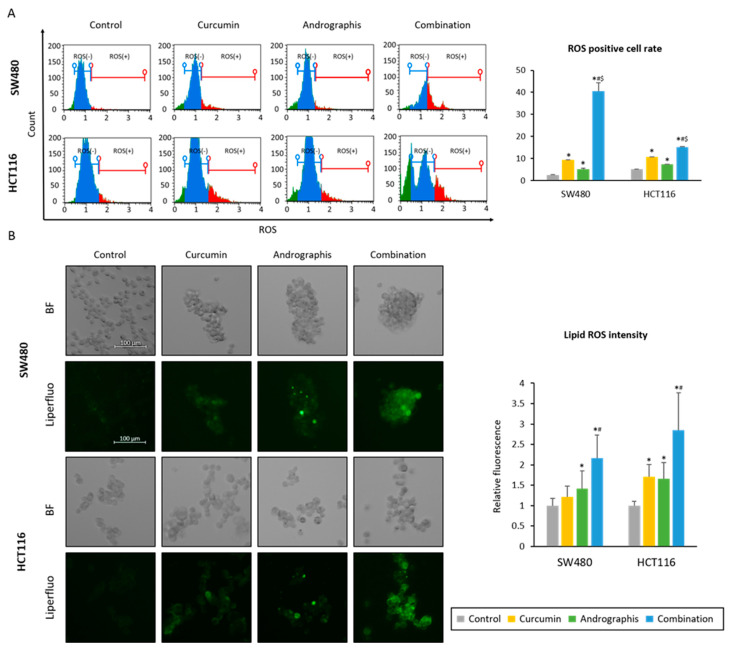
Curcumin and andrographis combination induced ferroptosis via intracellular accumulation of ROS and lipid peroxidases. (**A**) Representative images of the cells undergoing oxidase stress in SW480 and HCT116. The average (column) ± SD is indicated (*; *p*-value < 0.05 vs. control, #; *p*-value < 0.05 vs. curcumin, $; *p*-value < 0.05 vs. andrographis). (**B**) Representative images of the Liperfulo assay in SW480 and HCT116 following treatment with curcumin, andrographis, and their combination for 16 h using a fluorescent microscope under 200× magnification with a 488 nm/550 nm excitation filter (scale bar = 100 μm). The average (column) ± SD is indicated (*; *p*-value < 0.05 vs. control, #; *p*-value < 0.05 vs. curcumin). Cur: curcumin, andro: andrographis.

We examined whether the curcumin and andrographis combination could induce the accumulation of reactive oxygen species (ROS) in CRC cells. First, we read the intracellular ROS accumulation induced by each treatment. The ROS-positive cell population in the control group was 2.6% in SW480 and 5.3% in HCT116 cell lines (Figure 4A). Curcumin and andrographis treatments significantly increased the ROS-positive cell population (curcumin, 9.5% in SW480, 10.8% in HCT116, and andrographis, 5.2% in SW480, 7.4% in HCT116, Figure 4A). Moreover, the combination treatment dramatically increased the ROS-positive cell population in both cell lines (40.6% in SW480 and 15.2% in HCT116; Figure 4A).

Secondly, as a downstream event of ROS accumulation, we evaluated the changes in lipid peroxidation levels using live cell imaging (Figure 4B). By curcumin mono treatment, relative lipid peroxidation levels compared to the untreated controls were 1.2 times higher in SW480 (*p* = 0.07) and 1.7 times higher in HCT116 cell lines (*p* < 0.05). Similar to curcumin, andrographis treatment also showed significantly higher lipid peroxidation levels vs. control, which were 1.4 times higher in SW480 (*p* < 0.05) and 1.7 times higher in HCT116 (*p* < 0.05). Furthermore, curcumin and andrographis combined treatment showed more than twofold higher lipid peroxidation levels compared to the controls in both cell lines (relative lipid peroxidation level, 2.1 in SW480; 2.8 in HCT116). These results and pathway analysis revealed that curcumin and andrographis combination treatment could modulate the ferroptosis pathway and, as a result, induce ferroptosis, an iron-dependent apoptotic process, in CRC cells.

These results and pathway analysis revealed that curcumin and andrographis combination therapy could modulate the ferroptosis pathway and, as a result, induce ferroptosis in CRC cells.

### 2.4. Ferroptosis Suppressor, Ferrostatin-1, Reversed the Anti-Tumor Effects of Curcumin and Andrographis Combination in Cancer Cells and Patient-Derived Organoids

**Figure 5:**
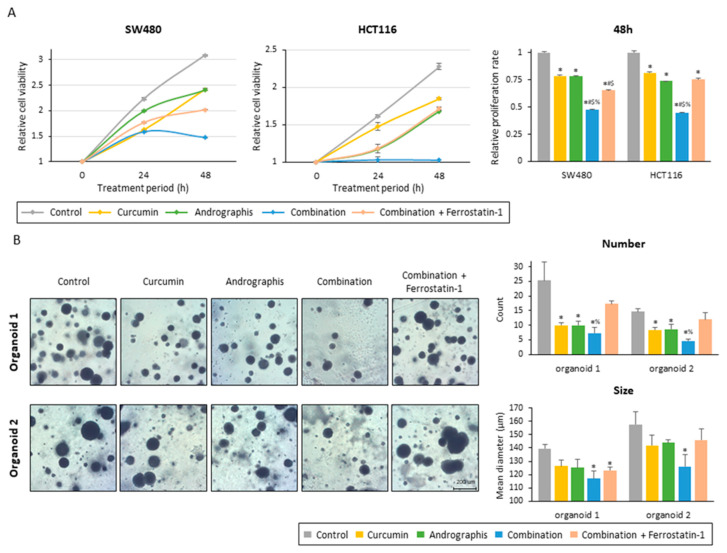
Ferrostatin-1 reversed the anti-tumor effect of the curcumin and andrographis combination. (**A**) Cell proliferation assay in SW480 and HCT116 following treatment with curcumin, andrographis, their combination, and combination plus ferrostatin-1. The error bars represent the mean ± SD. The average (column) ± SD is indicated (*; *p*-value < 0.05 vs. control, #; *p*-value < 0.05 vs. curcumin, $; *p*-value < 0.05 vs. andrographis, %; *p*-value < 0.05 vs. combination). (**B**) Representative images, counts, and sizes of patient-derived organoids following treatment with curcumin, andrographis, their combination, and ferrostatin-1. The average (column) ± SD is indicated (*; *p*-value < 0.05 vs. control, %; *p*-value < 0.05 vs. combination). Cur: curcumin; andro: andrographis; Fer-1: ferrostatin-1.

To examine whether the ferroptosis-inducive potential of this combination treatment can be responsible for the anti-tumor growth effects in CRC cells and patient-derived organoids, we included ferrostatin-1, an inhibitor of ferroptosis, along with the combination treatment. The proliferation assay of two colon cancer cell lines showed that ferrostatin-1 significantly reversed the anti-proliferation effects of the combined treatment with the two botanicals in both cancer cells (Figure 5A). By inhibiting ferroptosis, relative proliferation rates were recovered from 0.47 to 0.65 for SW480 (*p* < 0.01) and from 0.45 to 0.75 for HCT116 (*p* < 0.01; Figure 5A).

Secondly, we evaluated the effects of the combined treatment on patient-derived 3D organoids. Similar to the cancer cells, this combination dramatically suppressed the growth of organoids, and as a result, the number and mean size of organoids were significantly reduced (Figure 5B). The combination treatment decreased the average number of organoids per field from 25.3 to 7.3 in organoid 1 (control vs. combination, *p* < 0.01) and from 14.7 to 4.7 in organoid 2 (control vs. combination, *p* < 0.01; Figure 5B). The mean size of organoids was also decreased by this combined treatment, from 140 µm to 117 µm for organoid 1 (*p* < 0.01) and from 158 µm to 126 µm for organoid 2 (*p* < 0.05) (Figure 5B). These results showed that the combined treatment with curcumin and andrographis showed anti-tumor effects in cancer cells and patient-derived CRC organoids that more closely reflect the tumor microenvironment. Furthermore, using ferrostatin-1, reversed the impact of the combination, especially regarding the number of organoids. The average number of organoids for the ferrostatin-1 treated group was 17.3 for organoid 1 (combination vs. ferrostatin-1, 7.3 vs. 17.3, *p* < 0.05) and 12.0 for organoid 2 (combination vs. ferrostatin-1, 4.7 vs. 12.0, *p* < 0.01), which is significantly higher than the combination and comparable to the control (Figure 5B). Collectively, these results highlight that curcumin and andrographis combination treatment exhibited anti-tumor effects on patient-derived organoids. The ferroptosis-inducive potential of this combination might be responsible for their anti-cancer efficacy in CRC cells and tumor-derived organoids.

## 3. Discussion

Colorectal cancer (CRC) is the third most common malignancy and the second leading cause of cancer-related deaths worldwide [1]. More than 20% of patients have distant metastasis disease at diagnosis, and their 5 year survival rate is less than 15% [3,4]. For such patients, systemic chemotherapy is still the primary treatment option [4]. Limitations of current therapies include their toxicity, side effects, and cost-effectiveness. Several naturally occurring compounds have been considered important to assess these unmet needs in metastatic CRC treatment because of their multi-targeted efficacy and time-tested safety compared to conventional drugs [5,7,8]. Ferroptosis is a unique type of programmed cell death depending on the intracellular accumulation of iron, followed by enhanced production of reactive oxygen species (ROS) and lipid peroxides [5,18,19]. Recently, ferroptosis has been considered an endogenous anticancer mechanism and is actively being studied to develop novel therapeutic targets in cancer [5]. Previous studies reported that natural products, including curcumin and andrographis, might induce ferroptosis in cancer cells [5,16,20,21,22]. Thus, we hypothesized that a combination treatment of curcumin and andrographis might further enhance their anti-tumor potential via ferroptosis.

The current study found that combining curcumin and andrographis was superior to either compound alone. The combined treatment with these compounds showed promising anti-tumor effects in cancer cells and patient-derived organoids. Moreover, genome-wide expression profiling analysis and subsequent assays revealed that the anti-tumor effects of the combined treatment were at least partially mediated via the induction of ferroptosis through the dual suppression of GPX-4 and FSP-1. In the current study, in line with previous studies, monotherapy with curcumin and andrographis showed promising anti-tumor effects via induction of apoptosis. However, in this present study, the co-administration of curcumin and andrographis synergistically exhibited promising anti-tumor growth effects, especially ferroptosis induction, which was much stronger than curcumin or andrographis alone. Therefore, this combination treatment resulted in cancer cell death via both forms of cell death: apoptosis and ferroptosis.

GPX-4 is a primary regulator suppressing ferroptosis and also reported as a potent prognostic factor in various malignancies, including CRC [29,30,31,32,33]. Therefore, GPX-4 inhibition is considered to be a promising pharmacological target. However, in some cancer cell lines, suppression of GPX-4 cannot trigger ferroptosis, suggesting that alternative ferroptosis-resistant modulators exist in cancer cells [27,28,34]. In line with this hypothesis, more recently, FSP-1, previously called apoptosis-inducing factor mitochondria associated 2, was identified as a GPX-4 independent ferroptosis suppressor, and pharmacological FSP-1 inhibition strongly synergized with a GPX-4 inhibitor to induce ferroptosis in many cancer cells [27,28]. Thus, dual suppression of GPX-4 and FSP-1 is considered a promising approach to cancer treatment. Accordingly, an important mechanistic aspect of the curcumin and andrographis combined treatment is the dual suppression of GPX-4 and FSP-1, which has the potential to overcome the resistance of current chemotherapies and be beneficial to CRC patients in clinical practice.

There are a few potential limitations to our current study. We did not perform pre-clinical animal studies to study the effects of the two compounds. Instead of animal experiments, we performed patient-derived organoid experiments to reveal the anti-tumor effects of curcumin and andrographis and their combination. Patient-derived tumor organoids have received increasing attention as novel ex vivo models for studying anti-cancer drug screening [35,36,37,38]. While 2D in vitro models are widely used for drug screening owing to their accessibility, these models do not reflect tumor heterogeneity and the tumor microenvironment [37,39]. Organoids are considered to reflect tumor genetic diversity and microenvironment [39]. Therefore, organoids offer a promising platform to evaluate the efficacy of various anti-tumor drugs and compounds. Some previous reports have shown that organoids might offer an alternate and comparable experimental platform compared to animal models [36,39]. Accordingly, we utilized an organoid model to validate our cell culture findings, and the results were comparable in both experimental models. However, the detailed genomic characterization of the organoids was not undertaken because the primary purpose of our experiments was not to focus on specific cancer genotypes. In addition, we will also plan to validate our findings and evaluate the potential toxicity of this combination using in vivo animal models in the near future.

## 4. Materials and Methods

### 4.1. Cell Lines and Reagents

Human CRC cell lines (HCT116 and SW480) were obtained from the American Type Culture Collection (ATCC, Manassas, VA, USA). Both cell lines were cultured in Dulbecco’s modified Eagle’s medium (DMEM; Gibco, Carlsbad, CA, USA) supplemented with 10% fetal bovine serum (Gibco, Waltham, MA) and 1% penicillin-streptomycin. All cells were maintained in a 37 °C incubator with a 5% humidified CO2 atmosphere, and the cell culture medium was replenished every two days. Ferrostatin-1 (Sigma-Aldrich, MO, USA) was dissolved in dimethyl sulfoxide and diluted to appropriate concentrations in culture media.

### 4.2. Herbal Preparations

Curcumin (BCM-95, Arjuna Natural Extracts, India, and EuroPharma USA, Green Bay, WI) and andrographis (EP80; EuroPharma USA, Green Bay, WI) were used in this study. BCM-95 is a curcumin complex extracted from *Curcuma longa* and standardized to 86% curcuminoids and 7–9% essential turmeric oils. Andrographis was extracted from the leaves of *Andrographis paniculata*. The extract was dissolved in 70% ethanol and standardized for 20% andrographolide content. Both compounds were dissolved in dimethyl sulfoxide and diluted to appropriate concentrations in culture media.

### 4.3. Cell Viability Assays

For these assays, five thousand cells were seeded in 96-well flat plates. After twelve hours of cell seeding to allow cell attachment to the plates, each cell line was treated with curcumin, andrographis, or their combination using different concentrations for forty-eight hours. After treatment, the cell viability assays were performed using the Cell Counting Kit-8 (CCK-8) kits (Dojindo, Kumamoto, Japan). A quantity of 10 μL of the CCK-8 reagent was added to each well, and the cells were incubated in a 37 °C incubator for two hours. After that, the absorbance of the cells was analyzed at 450 nm using an enzymatic plate analyzer (Molecular Devices, San Jose, CA, USA).

### 4.4. Invasion Assays

The method for these assays was previously described [10,21]. CRC cell lines were seeded in 6-well plates and treated with compounds for forty-eight hours. After the treatment, 2.5 × 104 cells were seeded into the Matrigel invasion chamber (Corning, Tehama County, CA, USA) with an 8.0 µm PET membrane. Diff-Quick was used to fix and stain invaded cells 48 hours after the invasion began.

### 4.5. Colony Formation Assays

Colony formation assays were performed as previously reported [23,40]. Five hundred cancer cells were seeded in 6-well plates. After 3–5 days of incubation, cells were treated with individual compounds or their combinations for forty-eight hours. Following the completion of the treatment period, cells were cultured in a standard culture medium for another week and subsequently harvested. The harvested cells were fixed to cell culture plates with 100% methanol for twenty minutes and stained with 1% crystal violet overnight. As previously reported, colony formation rates were quantified as the percentages of the stained area using ImageJ [41].

### 4.6. RNA-Sequencing and Pathway Analysis

For these experiments, 2.5 × 10^5^ cells from SW480 and HCT116 lines were treated with each compound for 16 h before total RNA extraction using the MiRNeasy Mini kit (Qiagen, Hilden, Germany). The details for RNA-sequencing library preparation were previously reported [12,23,40]. Briefly, the library was constructed using the TruSeq RNA Sample Prep Kit (Illumina, San Diego, CA) with 1 μg of purified RNA input. Thereafter, the size and concentration of purified library products were assessed using a Bioanalyzer DNA High Sensitivity Kit (Agilent Technologies, Santa Clara, CA). Paired-end sequencing (150 bp on each end) of the validated library products was then analyzed on the HiSeq X-Ten system (Illumina). All sequencing data have been deposited in the GEO database (GSE223195).

For sequencing data analysis, differentially expressed genes (DEG) between treated and untreated cells were extracted using a threshold of *p* < 0.05 and |log2 fold changes| > 0.5. Gene Ontology and KEGG enrichment pathway analysis were performed on all DEGs to determine the most influential pathway.

### 4.7. Real-Time Quantitative Reverse Transcription PCR (RT-qPCR) Assays

Total RNA was extracted from cancer cells treated for 16 h. Using a high-capacity cDNA reverse transcription kit (Thermo Fischer Scientific, Waltham, MA, USA), RNA was reverse transcribed to complementary DNA (cDNA). The qRT-PCR assays were performed using a SensiFAST SYBR Lo-ROX Kit (Bioline, London, United Kingdom) and the QuantStudio 6/7 Flex RT-PCR System (Applied Biosystems, Foster City, CA, USA). GAPDH was used as the housekeeping gene, and gene expression changes were calculated by the delta-Ct method. The primer sequences used in this study are described in Table S1.

### 4.8. Western Blotting

Western blotting was performed as previously reported [23,24,40]. Cells were treated with each compound for sixteen hours to purify proteins and subsequently lysed using the RIPA buffer with a proteinase inhibitor cocktail (ThermoFisher Scientific, Waltham, MA, USA). Purified protein extract was denatured with 2X Laemmli’s sample buffer (Bio-Rad Laboratories, Hercules, CA, USA), which contained 5% 2-mercaptoethanol (Sigma–Aldrich). The list of primary and secondary antibodies is described in Table S2. The GAPDH was used as a loading control, and band intensities were quantified using Image J software.

### 4.9. Apoptosis and Oxidase Stress Assays

Apoptosis and oxidative stress assays were performed after a 16 h cell line treatment. Per the manufacturer’s instructions, these assays were performed using the Annexin V and Dead Cell Kit and the Oxidative Stress Kits on the Muse Cell Analyzer (Millipore Corp., Billerica, MA).

### 4.10. Lipid Peroxidase Assays

As per the manufacturer’s protocol, lipid peroxidase levels were evaluated using Liperfluo reagent (Dojindo, Kumamoto, Japan). Cancer cells were seeded in 12-well plates and treated for 16 h. After the treatment period, cells were incubated with 10 μM Liperfluo for thirty minutes at 37 °C. After that, cells were gently washed twice with PBS, and fluorescence images were obtained using a confocal microscope.

### 4.11. Patient-Derived Tumor Organoids

Two patient-derived CRC organoids were developed and cultured as previously reported [12,22,23,24,40]. Briefly, surgically resected tumors were maintained in DMEM-F12 (Gibco) supplemented with 1% HEPES (Sigma-Aldrich), 1% L-glutamine (Gibco), 10% FBS (Gibco), 2% penicillin/streptomycin (Sigma-Aldrich), and 10 μM Y-27632 (R&D Systems). Tumors were digested with collagenase solution (5 mL of the above medium with 75 μL collagenase, 124 μg/mL dispase type II, and 0.2% Primocen) for 30 min and then filtered through a 70 μm filter (Corning). An organoid pellet was obtained by centrifugation (200× *g* for 10 min). Organoids were suspended in Matrigel (Corning, Tehama County, CA) with IntestiCult™ Organoid Growth Medium (#06010, STEMCELL Technologies) and seeded in 12-well plates. Approximately 750 μL of IntestiCult™ Organoid Growth Medium was added to each well. Organoids were divided into five groups of control, curcumin (3.0 μg/mL), andrographis (30.0 μg/mL), their combination (curcumin; 3.0 μg/mL, andrographis; 30.0 μg/mL), and their combination plus ferrostatin-1 (curcumin; 3.0 μg/mL, andrographis; 30.0 μg/mL; ferrostatin-1; 20 μM). Following forty-eight hours of treatment, the numbers of organoids (<100 μm) and their mean sizes were examined using Image J software.

According to the Declaration of Helsinki, written informed consent was obtained from all patients, and the institutional review board approved all experiments at the institution. Clinical features of CRC patients for organoid isolation are described in Table S3.

### 4.12. Statistical Analysis

All assays were performed in triplicate, and the data are described as the mean ± standard deviation. Statistical comparisons among each treatment group were made using the Tukey–Kramer method, and a *p* value of < 0.05 was considered to represent statistical significance. All analysis was conducted using JMP 8.0.1 (SAS Institute Inc., Tokyo, Japan).

## 5. Conclusions

In conclusion, we first demonstrated that a novel combination treatment regimen consisting of curcumin and andrographis exhibited anti-tumorigenic effects in CRC through ferroptosis caused by dual suppression of GPX-4 and FSP-1. Our findings could provide essential evidence for the curcumin and andrographis combination as a potential clinical application for patients with CRC.

## Data Availability

Data is contained within the article and supplementary material.

## Supplementary Materials

The following supporting information can be downloaded at: https://www.mdpi.com/article/10.3390/ph16030383/s1, Table S1: List of primers for qPCR; Table S2: List of antibodies for Western blotting. Table S3: Clinical features of CRC patients used for the organoid establishment.
