# Supplementary material for: Curcumin and Andrographis Exhibit Anti-Tumor Effects in Colorectal Cancer via Activation of Ferroptosis and Dual Suppression of Glutathione Peroxidase-4 and Ferroptosis Suppressor Protein-1
Source: Pharmaceuticals (Basel). 2023 Mar 2;16(3):383. doi: 10.3390/ph16030383 (PMC10055708; doi:10.3390/ph16030383)
Supplement: Supplementary file 1 [file pharmaceuticals-16-00383-s001.zip › pharmaceuticals-2232974-supplementary.pdf]

Table S1. List of primers for qPCR.

**Gene**	**Forward**	**Reverse**
GPX-4	ACAAGAACGGCTGCGTGGTGAA	GCCACACACTTGTGGAGCTAGA
FSP-1 (AIFM-2)	AGACAGGGTTCGCCAAAAAGA	CAGGTCTATCCCCACTACTAGC
GAPDH	TGCACCACCAACTGCTTAGC	GGCATGGACTGTGGTCATGAG

Table S2. List of antibodies for western blotting.

**Target**	**ID**	**Company**
GPX-4	MAB5457	R&D Systems, MN, USA
FSP-1(AIFM-2)	20886-1-AP	Proteintech Group Inc, IL, USA
GAPDH	10494-1-AP	Proteintech Group Inc, IL, USA
Anti-rabbit IgG	#7074	Cell Signaling Technology, MA, USA
Anti-mouse IgG	#7076	Cell Signaling Technology, MA, USA

Table S3. Clinical features of CRC patients used for the organoid establishment.

ID	Age	Sex	Ethnicity	Location	Tumor depth	Lymph node metastases
Organoid 1	67	Female	Caucasian	Cecum	T4b	N1b
Organoid 2	40	Male	Caucasian	Rectum	T3	N0
